# Zhenzhu Xiaoji Decoction Induces Autophagy and Apoptosis Cell Death in Liver Cancer Cells through AKT/mTOR and JAK2/STAT3 Signaling Pathway

**DOI:** 10.1155/2022/4445293

**Published:** 2022-04-06

**Authors:** Yang Sun, Yue Sun, Songzhe Li, Xuelian Tao, Lingyun Cai

**Affiliations:** Department of Biology, College of Basic Medicine, Heilongjiang University of Chinese Medicine, Harbin 150040, Heilongjiang, China

## Abstract

**Background:**

Liver cancer is one of the most common digestive tumors. The prescription Zhenzhu Xiaoji decoction (ZZXJD) has a certain effect on the growth and survival of primary liver cancer. *Object*: This article aimed to explore the effect and molecular mechanism of ZZXJD on liver cancer SMMC-7721 cells.

**Method:**

The research groups were divided into the model group, ZZXJD group, and cisplatin group. SMMC-7721 cells were treated with different concentrations of ZZXJD-medicated serum for 24 h and 48 h. The cell viability was measured with CCK8 assay, and cell morphology was observed by fluorescence microscope and transmission electron microscope (TEM). Western blot, RT-PCR, and gene chip were used to determine the protein expression level and gene expression level of cells and tumor tissues.

**Results:**

ZZXJD inhibited the proliferation activity of SMMC-7721 cells in a concentration- and time-dependent manner. The morphological changes of the cell showed apoptosis and autophagy. The gene expression of protein kinase B (AKT), mammalian target of rapamycin (mTOR), Janus kinase 2 (JAK2), and signal transducer and activator of transcription 3(STAT3) were downregulated compared with the model group(*p* < 0.05). The nude mice experiments confirmed that ZZXJD inhibited the growth of tumors in tumor-bearing mice, and the effect increased with the increase of concentration.

**Conclusion:**

ZZXJD induced autophagy and apoptosis of liver cancer cells via inhibiting AKT/mTOR signaling pathway and JAK2/STAT3 signaling pathway, thereby affecting the growth and survival of liver cancer cells.

## 1. Introduction

Liver cancer is currently one of the most common malignant tumors. The incidence rate of liver cancer is increasing year by year. According to the survey report in 2018, the incidence rate accounted for 4.7% of malignant tumors, and the mortality rate is 8.2% of malignant tumors [1]. In 2019, a research report pointed out that the mortality rate had accounted for 9.1% [2]. At present, the treatment of liver cancer focus on noncoding RNA and immunotherapy, while the long-term efficacy of monotherapy is not ideal, and the interaction between the mechanisms of combination therapy is unclear [3, 4]. Lots of research showed that traditional Chinese medicine (TCM) had inhibitory effects on tumor growth, proliferation, migration, and metastasis. Integrated TCM and other treatments can reduce adverse reactions and prolong the survival of patients. It can be taken throughout the course of treatment [5]. The focus of current research is to find effective TCM and to fully define its mechanism of action. The JAK/STAT signaling pathway can be abnormally activated by cytokines and other biological processes, such as cell proliferation, differentiation, autophagy, apoptosis, and immune regulation. It is an important signaling pathway for the occurrence and development of tumors and other diseases [6]. The mTOR signaling pathway can participate in the occurrence and development of tumors by inhibiting tumor cell apoptosis or cell autophagy [7, 8]. The two signaling pathways are both hot spots in tumor research.

The prescription ZZXJD is composed of *Ligustrum lucidum* (*Ligustrum lucidum Ait., fruit, nvzhenzi*), *Curcuma zedoaria (Curcuma phaeocaulis Val, root, ezhu)*, *Hedyotis diffusa (Spreading Hedyotis Herba, baihuasheshecao)*, *Prunella vulgaris(Prunella vulgaris* L.*, cluster, xiakucao)*, and *Licorice(Glycyrrhiza uralensis Fisch.*, *root, gancao*) [9–12]. Our preliminary research found that it had a certain inhibitory effect on growth and development of H22 liver cancer cell. Lots of autophagosomes were observed by TEM in ZZXJD group [13]. This experiment explored the effect of ZZXJD on human liver cancer SMMC-7721 cells and its molecular mechanism in *vivo* and in *vitro*, which provided new ideas and experimental basis for the research of new anticancer drugs and clinical medications.

## 2. Materials and Instruments

### 2.1. Materials and Animals

Human liver cancer SMMC-7721 cells were purchased from Fuheng, Shanghai, China. SD rats were identified and provided by Liaoning Changsheng Biotechnology company (Qualified number: SCXK-Liao-2020-0001). Sixty six-to-eight-week-old SD rats (240 ± 20 g) were half male and half female. BALB/*c* nude mice, provided by Beijing Weitong Lihua Laboratory Animal Technology (qualified number: SCKK-Jing-2016-0006), which were six-to-eight weeks old (15 ∼ 20) ±2 g weight and a total of 60 males and females. The experiment and breeding were carried out in a barrier environment, and the water and feed are sterilized. The animal study was reviewed and approved by Heilongjiang University of Chinese Medicine Institutional Animal Care and Use Committee (no. 2019051301; 2019112201). ZZXJD was provided from the First Affiliated Hospital of Heilongjiang University of Chinese Medicine (Table 1). The herbs were authenticated by a pharmacognosist from the Heilongjiang University of Chinese Medicine, in accordance with standard protocols of the Chinese Pharmacopoeia (Version 2020). The ingredients of the Chinese medicine have been tested and identified. The autophagy inhibitor was 3MA (Abmole, M2296), and the positive control drug was cisplatin (Abmole, M2223).

### 2.2. Instruments

The following instruments were purchased: experimental instrument PrimeScript TM RT Reagent Kit (for real time) (TAKARA company), SDS electrophoresis instrument (Bio-Rad, 042BR13724), microabsorption instrument (Implen, p330), membrane transfer instrument (Bio-Rad, 221BR52130), vertical gel electrophoresis tank (Bio-Rad, 552BR113758), gel imaging system (Bio-Rad, Universal hood II), decoloring shaker (Wode Biomedical Instruments, WD-9405B), automatic image analysis system (TANON, 5020), transmission electron microscope (HITACHI, H-7650), autoclave (Panasonic, MLS-3751L) -PC), pure water system (MILLIPORE, TANKPE0600), and electric heating blast drying oven (Tianjin Test Instrument Co., Ltd., WGL-65B).

## 3. Method

### 3.1. Cell Culture

The DMEM complete culture medium (Boster, #PYG0103) is added with 10% FBS and 1% penicillin/streptomycin. SMMC-7721 cells were observed by inverted microscope. When the cells placed under the culture flask are about 70 ∼ 80%, the original culture medium was discarded, and the culture flask was gently rinsed with PBS. Then, trypsinization was added in the flask. When the intercellular space of cells enlarged, fresh culture medium was put in the flask to finish digestion of trypsinization. After pipetting and centrifugation, the culture medium was extracted carefully on the cell upper layer, and then, the cells were made a single cell suspension by the fresh culture medium. The cells were cultured at 37°C with 5% CO_2_.

### 3.2. Preparation of Medicated Serum

SD rats were bred adaptively in a sterile environment for three days, and the changes in physiological conditions such as body weight, food intake, and hair of the rats were observed. The dose of ZZXJD was 65 g·kg^−1^ body weight, which was calculated according to the equivalent dosage table of human and rat body surface area. The rats were administered by gavage for 3 days, and blood was taken from the abdominal aorta to prepare medicated serum.

### 3.3. CCK8 Method

The mixed single cell suspension was diluted with complete culture medium to 5 × 10^4^ cells/mL and inoculated in a 96-well plate at 100 *μ*L/well. The outer circle of the well plate was PBS solution, and the well plate was moved to 37°C, 5% CO_2_ for 24 h. The supernatant was discarded, and the plate was washed twice with PBS. Then, 100 *μ*L culture medium with ZZXJD-containing serum (0, 2.5%, 5%, 7.5%, 10%, 12.5%, 15%, 17.5%, and 20%) was added to the plate, and each group set up six re-wells. After adding the medicine, cells were incubated for 24 h or 48 h, and 10 *μ*L CCK8 was added to each well solution, which was shaken and mixed and incubated for 2 h. OD value of each well was detected at 450 nm on a microplate reader. The proliferation activity of SMMC-7721 cells was calculated and analyzed. Cell viability was calculated by (experimental group OD value/control group OD value) × 100%.

### 3.4. Hoechst 33342 Staining

Cells were seeded at 1 × 10^4^ cells/well in 48-well plates for 24 h before treatment. The wells were added 0.25 mL culture medium with drug-containing serum of 0%, 5%, 10%, 15%, and 15% + 0.5 mM 3MA. The other two groups were 0.5 mM 3MA and 4 *μ*g/mL cisplatin. After adding the different medicine, the orifice plate was placed in the incubator for 24 h. After washing with PBS twice, Hoechst33342 staining solution 250 *µ*L was added to each well and placed in an incubator for 30 min. The morphology changes of SMMC-7721 cells were observed under fluorescent inverted microscope.

### 3.5. The Ultrastructure of Cell

Based on Hoechst 33342 staining result, cell ultrastructure experiment was carried out. Drug-containing serum 0% and 15% for 24 h were added to the cells. The cells were scraped by a cell scraper, fixed with 2.5% glutaraldehyde, washed with 0.1 M phosphate buffer, dehydrated through a graded ethanol series, followed by embedding, and imaged on H-7650 TEM (HITACHI, Japan).

### 3.6. Western Blot Assay

SMMC-7721 cells were treated with complete culture medium containing different concentrations of medicated serum (0, 5, 10, and 15%) and cisplatin (4 *μ*g/mL) for 48 h, centrifuged to remove the supernatant, and washed three times with PBS. An appropriate amount of cell lysate containing PMSF was added, and cells were lysed on ice. After quantification by the BCA protein quantification kit, GAPDH was used as an internal reference for Western Blot. Photoshop software, Image J, and GraphPad software were used to statistically calculate the data to analyze the expression of related autophagy proteins such as Beclin1, mTOR, and STAT3 in liver cancer SMMC-7721 cells. The level of protein and phosphorylation expression were measured.

### 3.7. qRT-PCR Assay

Cells treated with Cisplatin and different concentration ZZXJD-containing serum for 48 h. The total RNA of each group cells was extracted with Trizol (Invitrogen, USA). Use the NanoPhotometer™ P-Class ultramicro spectrophotometer to measure the light absorption values at 260 nm and 280 nm, and record the ratio of the two to determine the purity of the extracted RNA. The ratio of all test samples is required to be controlled between 1.8 and 2.1 to ensure the purity of the extracted RNA. Use PrimeScript First Strand cDNA Synthesis Kit to reverse-transcribe RNA into cDNA according to the instructions. Adopt SYBR premixed ExTaq kit (Takara, Dalian, China); PCR amplification was conducted at 35 cycles of denaturation at 95°C for 30 s, after predenaturation treatment at 95°C for 5 min, annealing treatment 58°C for 30 s and extension at 72°C for 30 s. The temperature at the end was 4°C. *GAPDH* was used as the internal reference gene to detect the relative expression levels of *Beclin1*, *mTOR*, *STAT3*, and other related genes. The primers of these gene were displayed in Table 2. Each sample was repeated three times to ensure the accuracy of the data. The relative expression levels of these genes were calculated in accordance with 2^−∆∆ct^.

### 3.8. Gene Chip

Gene chip experiments divided into two groups, which were the model group and 20% ZZXJT containing serum as drug group (noted drug and NC). The experiment was repeated three times. The RNA extraction was the same as the RT-PCR extraction. The total RNA extracted was tested by NanoDrop 2000 and Agilent Bioanalyzer 2100. The quality control standards were Thermo NanoDrop 2000 1.7< A260/A280 < 2.2, Agilent 2100 Bioanalyzer RIN ≥ 7.0 and 28S/18S > 0.7. The follow-up chip experiment was carried out after the sample was qualified. The signal intensity, principal component, and correlation analysis were based on chip data. Carry out significant difference analysis and statistical test on chip data qualified for quality control testing. Carry out functional and biological pathway analysis on differential genes. GraphPad Prism software, R language software for graphing analysis of related data, was used.

### 3.9. Tumor Suppression Experiment

BALB/c nude mice were randomly divided into five groups with 10 mice in each group. All nude mice were subcutaneously injected with 2.4 × 10^7^ SMMC-7721 cells resuspended in saline in the right axillary. A round bulge under the axillary was considered successfully after three days. Mice had palpable masses in the right axilla, and the tumor volume was about 45–50 mm^3^. Fifty nude mice were randomly divided into model, low of ZZXJD (16.25 g kg^−1^), medium dose of ZZXJD (32.5 g kg^−1^), high dose of ZZXJD (65 g kg^−1^), and cisplatin (5 mg kg^−1^ week^−1^) group. The model group nude mice were given an equal volume of normal saline. ZZXJD groups were given 0.2 mL of ZZXJD once a day. Cisplatin was intraperitoneally injected. The long and short diameters of the tumor were measured with a vernier caliper on the 3rd, 5th, 7th, 10th, 12th, and 14th days after inoculation. After 14 days, the tumor tissue was dissected and weighed. The tumor inhibition rate was calculated and analyzed.

### 3.10. Western Blot to Measure Protein Expression

Tumor tissue of different groups were grounded and crushed on ice to extract proteins. The proteins were separated by 120V voltage separation gel protein electrophoresis and 80V concentrated gel protein electrophoresis and then transferred to membranes and sealed at room temperature with TBST 1 h and added primary antibody (1 : 500–1 : 1000). GAPDH as internal control, GAPDH antibody (1 : 1000) was added and incubated overnight at 4°C. After washing three times with TBST on the second day, horseradish peroxidase, the second antibody (1 : 1000–1 : 25000), was incubated at room temperature for 1 h. TBST was washed again three times, and the electrochemiluminescence ECL kit was used to visualize the protein bands to reflect the expression level of each protein in the tissue. The experiment was repeated three times.

### 3.11. Statistics

GraphPad Prism 5 was used for statistical analysis, and the data were all expressed as $\bar{x}\pm s$. One-way analysis of variance, LSD, and SNK-q test were used for statistical analysis of data.

## 4. Results

### 4.1. The Effect of ZZXJD on the Proliferation of Liver Cancer SMMC-7721 Cells

**Figure 1:**
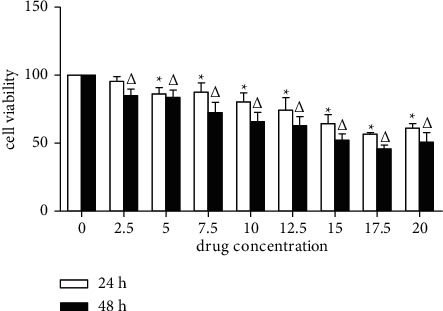
Cell viability (*X* ± *s*). Figure 1 shows that different doses of ZZXJD affected cell viability. ^*∗*^Compared with the model group, it is statistically significant (*p* < 0.05) in 24 h. ^Δ^Compared with the model group, it is statistically significant (*p* < 0.05) in 48 h.

To determine the antitumor effect of ZZXJD on liver cancer cells, the CCK8 assay was used to study the changes in cell viability. ZZXJD with different final concentrations was applied to SMMC-7721 cells for 24 h and 48 h. As shown in Figure 1 and Table 3, compared with the model group, the results of CCK8 at 24 and 48 h showed that 5–20% of ZZXJD had an inhibitory effect on SMMC-7721 cells, and the difference was statistically significant (*p* < 0.05). 5–17.5% of ZZXJD had the time- and concentration-dependence. The 24-hour result showed that 2.5% had an inhibitory effect on SMMC-7721 cells, but the difference was not statistically significant. It is worth noting that 20% ZZXJD has an inhibitory effect on SMMC-7721 cells at 24 h and 48 h (*p* < 0.05). But the inhibitory effect was less than 17.5%. As shown in Figure 1, after ZZXJD treatment for 24 h and 48 h, the cell viability of SMMC-7721 cells treated with ZZXJD decreased, and the inhibitory effect gradually increased with the increase of concentration and time at a certain concentration.

### 4.2. The Effect of ZZXJD on the Morphology of SMMC-7721 Cells

**Figure 2:**
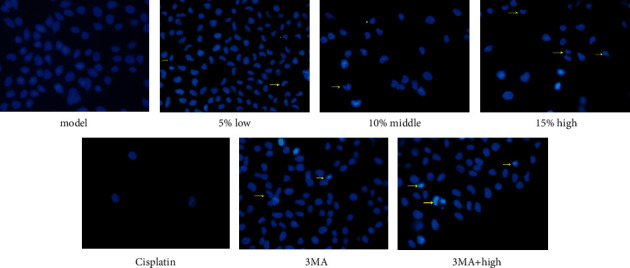
Fluorescence images of apoptotic morphology by Hoechst staining. The arrow represented the apoptosis cell. (a) Model, (b) 5% low, (c) 10% middle, (d) 15% high, (e) cisplatin, (f) 3MA, and (g) 3MA + high.

**Figure 3:**
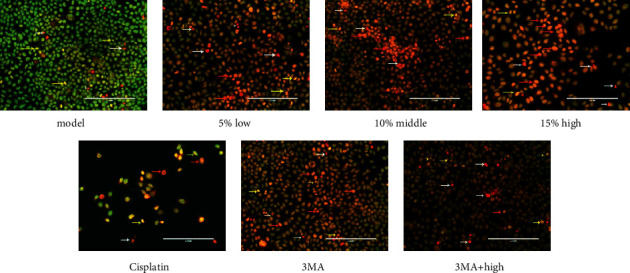
Fluorescence images of apoptotic morphology by AO/EB staining. The yellow arrow represented early-stage apoptosis cell. The red arrow represented late-stage apoptosis cell. The white arrow 295 represented apoptotic nonviable cells. (a) Model, (b) 5% low, (c) 10% middle, (d) 15% high, (e) cisplatin, (f) 3MA, and (g) 3MA + high.

**Figure 4:**
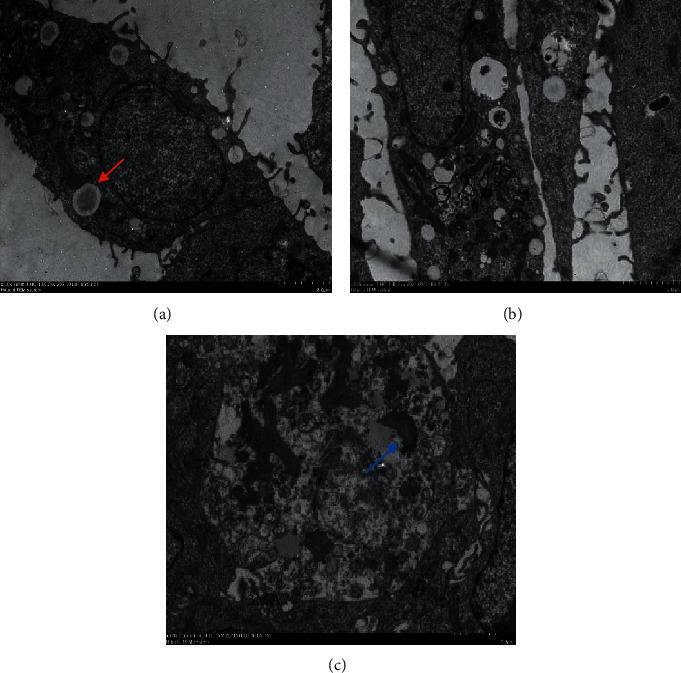
Effect of each drug group on cells under TEM. (a) is model group, and the red arrow represent lipid droplets. (b, c) Cell morphology of high-dose group. The yellow arrow represents autophagosome and the blue arrow point at the apoptosis body.

The Hoechst staining results are shown in Figure 2. Compared with the model group, the number of cells treated with drug decreased. In the meantime, the shape of cell nucleus changed into shrinkable and round, because the chromatin condensed into an irregular crescent shape. The cell nucleus disintegrated into fragments some time. Compared with ZZXJD group, ZZXJD + 3MA group and the cisplatin group had higher mortality of liver cancer cells. AO/EB double-staining distinguished apoptosis cells at different stages (Figure 3). The more cells were green AO nuclear staining in the control group. The cellular nucleus had condensed chromatin in early-stage apoptosis. The late-stage apoptosis cells, with increasing doses and asymmetrically localized orange nuclear EB staining, were also observed. The apoptotic nonviable cells are stained bright orange because of EB into these cells. In Figure 4, cellular structure of ZZXJD group (high dose; Figures 4(b) and 4(c)) was different to model control group (Figure 4(a)). The nuclear membrane of model group was integrated, whereas that of ZZXJD group was ruptured and the chromatin condensed in margin. In ZZXJD group, cell apoptosis and autophagy occurred at the same time, as the autophagosomes in Figure 4(b). Moreover, mitochondria were swelling and vacuolar degeneration.

### 4.3. The Effect of ZZXJD on Tumor Growth

**Figure 5:**
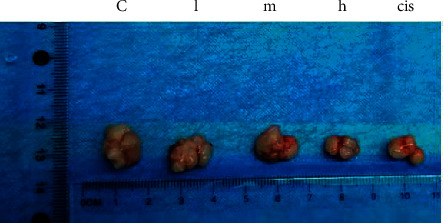
Tumor tissue of different group *c*: model control group; *l*: low-dose group; *m*: middle-dose group; *h*: high-dose group; cis: cisplatin group.

To explore the role of ZZXJD on tumor-implanted nude mice, the tumor weight and inhibition rate were analyzed. The results are shown in Figure 5 and Table 4. ZZXJD significantly decreased the tumor growth. The tumor inhibition rates of ZZXJD groups were 30.40%, 37.47%, 42.05%, and 42.05%, respectively. With the increase of the dosage, the tumor size gradually decreased, and the tumor inhibition rate gradually increased. The tumor inhibition rate of cisplatin group was 51.33%, and its tumor weighty was lighter than that of ZZXJD group (*p* < 0.05).

### 4.4. The Effect of ZZXJD on Protein Expression of Liver Cancer Cells

**Figure 6:**
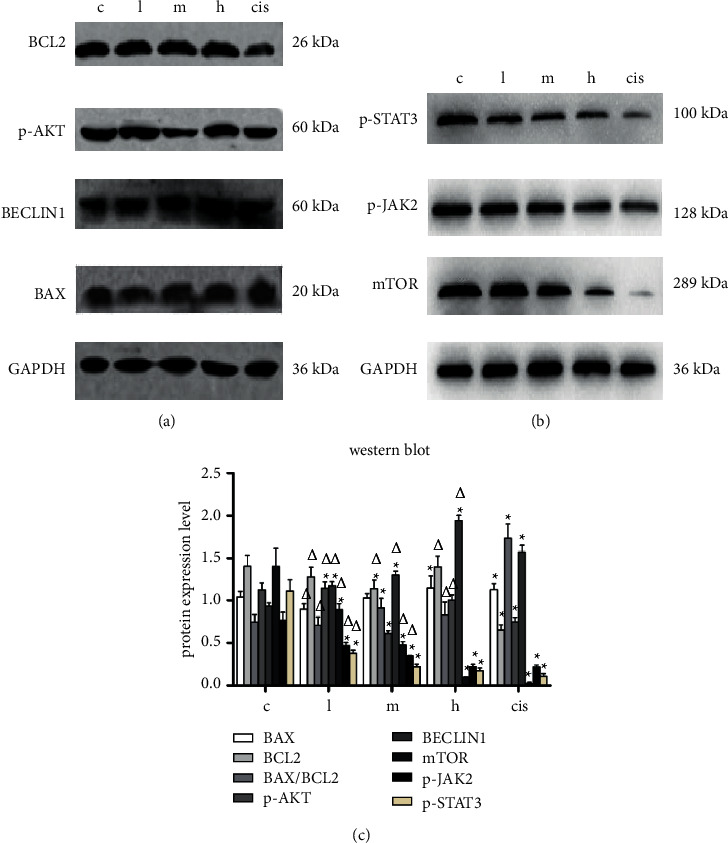
Proteins' expression in tumor of nude mice. ^*∗*^Compared with the model group, it is statistically significant (*p* < 0.05), and ^Δ^is statistically significant compared with the positive control group (*p* < 0.05).

Because cell apoptosis and autophagy occurred in the SMMC-7721 cells, the further in vivo experiment tested the related protein expression. The results of protein were shown in Figures 6(a)–6(c). Compared with the model group, BCL2 protein expression of the low-dose and high-dose ZZXJD group had no statistically significant difference (*p* > 0.05), while, in the middle-dose and cisplatin, it was downregulated, *p* < 0.05. BAX protein expression was upregulated in the high-dose ZZXJD and cisplatin group, *p* < 0.05. The middle-dose ZZXJD group had no statistically significant effect on the BAX protein but had statistical significance for the ratio of BAX/BCL2 ratio. BECLIN1 protein expression upregulates in the different drug groups, *p* < 0.05. About the possible mechanism, AKT/mTOR signaling pathway and JAK2/STAT3 signaling pathway were explored in the research. Compared with the model group, the p-AKT and mTOR protein expression were both downregulated (*p* < 0.05), except the p-AKT protein of low-dose group. JAK2/STAT3 signaling pathway key proteins (p-JAK2 and p-STAT3) were downregulated consistently.

**Figure 7:**
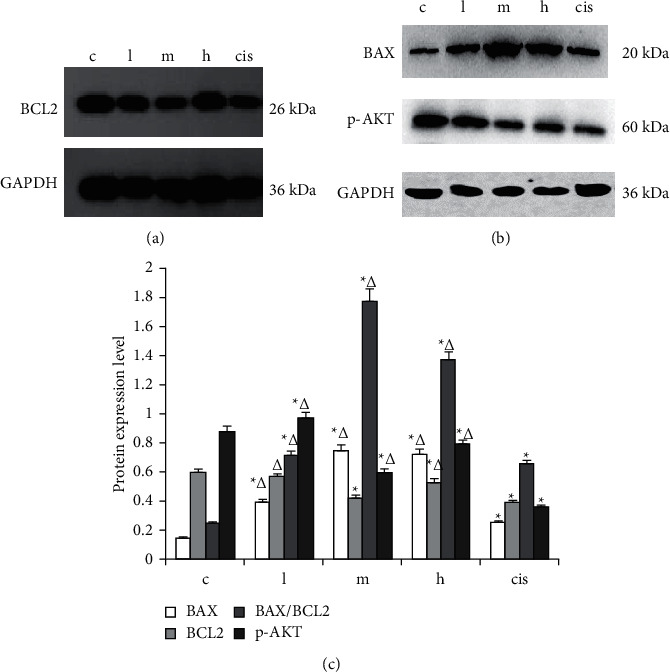
BAX, BCL2, and p-AKT protein expression of SMMC-7721 cell. ^*∗*^Compared with the model group, it is statistically significant (*p* < 0.05), and ^Δ^is statistically significant compared with the positive control group (*p* < 0.05).

Regarding the in vitro experiment, these protein expression analyses of SMMC-7721 cell treated with different drug were shown in Figure 7. Compared with the model group, BAX protein difference of ZZXJD group and cisplatin group was statistically significant (*p* < 0.05). BCL2 protein expression was downregulated in middle-dose ZZXJD group and cisplatin group. In terms of the protein expression of p-AKT compared with the model group, the difference in ZZXJD and cisplatin group was statistically significant (*p* < 0.05). For great clarity of autophagy role, there were the other two groups added in the further study, which were 3MA (autophagy inhibition) and 3MA + high-dose ZZXJD group. Compared with the model group, mTOR, p-JAK2, and p-STAT3 protein expression in the other six groups were downregulated (*p* < 0.05). Compared with cisplatin group, p-JAK2 and p-STAT3 protein expression had no statistically significant difference (*p* > 0.05), except mTOR (*p* < 0.05).

**Figure 8:**
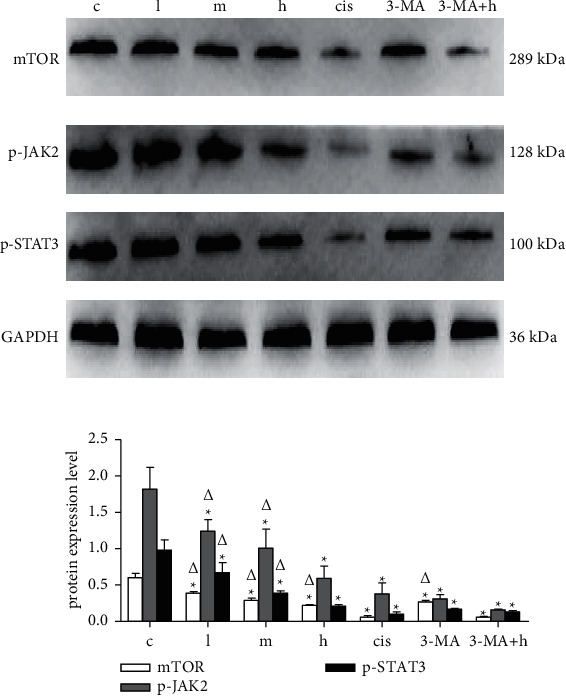
JAK/STAT/mTOR protein expression in SMMC-7721 cell $\left(\bar{X}\pm s\right)$, $\left(\bar{X}\pm s\right)$. ^*∗*^Compared with the model control group, it is statistically significant (*p* < 0.05), and ^Δ^is statistically significant compared with the positive control group (*p* < 0.05).

When the autophagy inhibitor 3 MA was used in combination with the high-dose ZZXJD, the difference in mTOR, p-JAK2, and p-STAT3 protein expression was statistically significant compared with the model group. Compared with cisplatin group, mTOR, p- JAK2, and p-STAT3 protein expression were not statistically significant (Figure 8).

### 4.5. The Effect of ZZXJD on Gene Expression Level of Liver Cancer Cells

#### 4.5.1. qRT-PCR to Detect Changes in the Level of Genes in Cells

**Figure 9:**
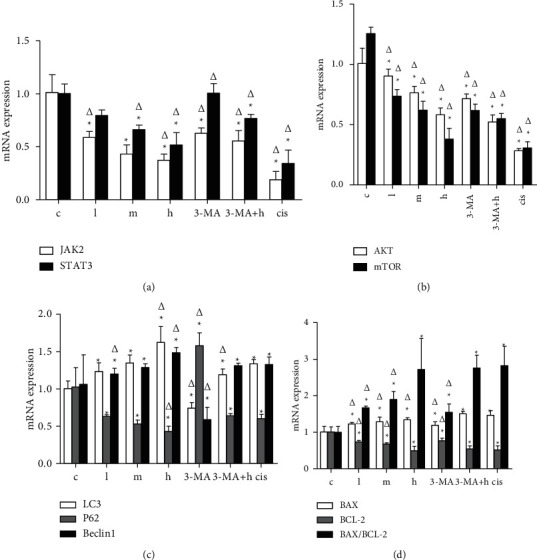
Gene expression in SMMC-7721 cells $\left(\bar{X}\pm s\right)$.^*∗*^Compared with the model group, it is statistically significant (*p* < 0.05), and ^Δ^is statistically significant compared with the positive control group (*p* < 0.05).

The liver cancer SMMC-7721 cells treated with drugs were analyzed by qRT-PCR. The results were shown in Figure 9. Compared with the model group, the autophagy and apoptosis-related genes detected in ZZXJD group were significantly changed (*p* < 0.05). As shown in Figure 9, *JAK2* and *STAT3* mRNA expression was gradually downregulated with the increase of the dosage of ZZXJD, and the difference was statistically significant compared with the model group and cisplatin group. When the autophagy inhibitor 3MA was used, *JAK2* mRNA expression was downregulated (*p* < 0.05), while *STAT3* mRNA was not statistically significant compared with the model group. In 3MA + high-dose ZZXJD group, *JAK2* and *STAT3* mRNA expression was upregulated (*p* < 0.05), which corresponded with protein expression results. As shown in Figure 9(b), *AKT* and *mTOR* mRNA expression in each drug group were statistically significant compared with the model control group, and the inhibition effect of ZZXJD was dose-dependent. The analysis of autophagy-related genes *LC3, P62*, and *BECLIN1* on SMMC-7721 cell line was shown in Figure 9(c). Compared with the model group, with the increase of the dose of ZZXJD, the autophagy-related genes *LC3* and *BECLIN1* were gradually upregulated, while *P62* mRNA was downregulated. When SMMC-7721 cells were treated with 3MA (autophagy inhibitor), the expressions of *LC3* and *BECLIN1* decreased (*p* < 0.05), while *P62* increased significantly (*p* < 0.01). In 3MA + high-dose ZZXJD group, *LC3* and *BECLIN1* mRNA increased, which suggested ZZXJD induced cell autophagy. The analysis of apoptosis-related genes in SMMC-7721 cell was shown in Figure 9(d). With the increase of the dose of ZZXJD, the mRNA expression of *BAX* and *BAX*/*BCL2* gradually increased. In contrast, *BCL2* mRNA expression declined compared with the model group (*p* < 0.05), and the high-dose ZZXJD group was not statistically significant compared with cisplatin. 3MA + high-dose ZZXJD group was similar to high-dose ZZXJD group, and *BAX/BCL2* ratio was consistent, too.

#### 4.5.2. Gene Chip

To analyze the whole transcriptome chip information of the collected sample gene chip, the quality of the original data of the chip was usually evaluated at first, and the subsequent information analysis was performed for the data that was qualified for quality control, including significant difference analysis and functional analysis of differential genes [14].

**Figure 10:**
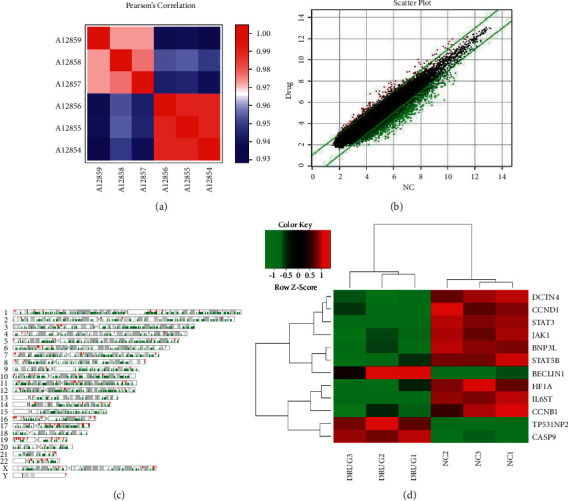
The results of gene chip (a) shows person's correlation between samples. (b) Scatter chart. (c) Differential gene chromosome distribution map. (d) Cluster analysis results.

As shown in Figure 10(a), the correlation analysis was accomplished through person correlation coefficients (PCC). PCC of drug and NC groups were both greater than 0.95, indicating that the gene expression trends of samples in the same group were highly similar. PCC between the two groups were significantly lower than PCC of the same groups, indicating large differences between them, which met the criteria for continued analysis.  Figure 10(b) was the scatter diagram of this gene chip, showing the distribution of signal intensity between the ZZXJD group and the model group on the rectangular coordinate system plane. The relatively parallel green solid line is the difference reference line, and the reference line is the points in the interval representing the probe groups with no significant changes. The red points outside the interval represent the probe groups that were relatively upregulated in the ZZXJD drug group (noted drug), and the green points represented the probe groups that were relatively upregulated in the model group (noted NC).  Figure 10(c) is a chromosome distribution map of differential genes, showing the distribution of differential genes on chromosomes and the specific positions of differential genes on each chromosome. The red in the figure represents the distribution of upregulated genes on the chromosomes, and the green represents the distribution of downregulated genes on the chromosomes.  Figure 10(d) is a heat map for hierarchical clustering of drug and NC samples with the expression profiles of differential genes screened by |fold change| ≥ 2.0 and FDR < 0.05 as the standard. Compared with the normal model group, *STAT3*, *STAT5B*, *JAK1*, and *IL6ST* were relatively downregulated in the medication group. The downstream genes *HIF1A* and *BNIP3L* of the JAK/STAT signaling pathway were downregulated, and the downstream *CASP9* of the AKT/mTOR signaling pathway was upregulated, which was positively correlated with autophagy. The genes *BECLIN1* and *TP53INP2* were relatively upregulated, while the gene *DCTN4* (P62), which is negatively related to autophagy, was relatively downregulated.

## 5. Discussion

Tumor is one of the most harmful diseases to human health, and it is also an important cause of the global burden of disease [15]. Excessive proliferation is one of the important malignant behaviors of tumor cells, so cell death is the research focus. Cell death plays an important role in being a stable balance by removing excess cells. Cell death concludes cell apoptosis (type I of programmed cell death, PCD), cell autophagy (type II of PCD), and necrotic programmed death (type III of PCD). Cell apoptosis is a continuous and dynamic process, which is the start of apoptosis, apoptosis body formation, and being engulfed by phagocytes. Cell autophagy is also a dynamic progress, which is the initiation, autophagosome formation, and being degraded in lysosome. Autophagy is a process of lysosome degradation of intracellular components, which is peculiar to eukaryotic cells. Autophagy is induced by external nutrients deficiency, ischemia, or hypoxia. The pressure also comes from cellular metabolic stress, aged or damaged organelles, and misfolded proteins. Autophagy and apoptosis are double-edged swords for cell growth and survival. Through the preliminary RNA-seq analysis, combined with the Western blot and qRT-PCR detection of this experiment, the research team found that the prescription ZZXJD inhibited the proliferation of liver cancer cells and was related to the PI3K/Akt/m TOR and JAK2/STAT3 signaling pathways.

PI3K/AKT/mTOR signaling pathway is an important signaling pathways involved in the progress of autophagy and apoptosis. mTOR is a receptor for amino acids and adenosine-triphosphate (ATP). Its activity is essential for the formation and maturation of autophagosomes. The mTOR pathway had confirmed that a variety of tumors had the feature of the pathway overactivation. Inhibiting this signaling pathway can induce cell cycle arrest, apoptosis, and autophagy. Moreover, it suppresses tumor metastasis and drug resistance [16]. The negative regulation of mTOR signaling pathway can activate ULK1 to phosphorylate it, thereby causing the formation of a series of autophagy complexes to induce autophagy. Conversely, inhibiting this pathway can induce autophagy [17]. STAT3 signaling pathway can be activated by cytokines and participates in all steps of the autophagy process, including autophagosome assembly and maturation. Its role is related to the stage of tumor development and the location of STAT3 protein. STAT3 protein with different locations plays different roles in cell's life activity. Autophagy is an important signal pathway for the occurrence and development of tumors and other diseases [6, 18]. After tumor-implanted nude mice were treated with different dose of ZZXJD or cisplatin, the cell proliferation was suppressed significantly compared with the model group. Combined with the in vitro SMMC-7721 cell experiment, ZZXJD and cisplatin both downregulated the gene and protein expression of AKT/mTOR and JAK2/STAT3 and regulated the gene expression of apoptosis- and autophagy-related gene expression, such as BAX, BCL-2, and BECLIN1 [19–21]. BECLIN1 is the first discovered key factor related to autophagy regulation. BECLIN1 can form a complex with III-type PI3K and mobilize autophagy-related proteins to localize to the proautophagosome. BAX and BCL2 are both important members of the BCL2 family, where BCL2 family plays an apoptotic role mainly by regulating the opening of mitochondrial membrane channels and the flow of proapoptotic substances. BAX is a key protein in the process of cancer apoptosis, which can promote the apoptosis of cancer cells. BCL2 has the opposite effect of BAX. It is a key protective protein for cancer cells and can inhibit cancer cell apoptosis [22]. The combination of BAX and BCL2 can form an apoptotic dimer, promote the release of apoptosis-inducing factors, and cascade with caspase protein to induce apoptosis [23]. BAX and BCL2 belong to BCL2 family and are involved in the mitochondrial apoptosis [24]. BCL2 not only played an important role in apoptosis, but also in autophagy. LC3 is a marker protein for detecting autophagy and is the first autophagosome membrane protein. In the process of autophagy, LC3 is cleaved by ATG4 with endoproteinase activity to produce LC3-I, LC3-I. Ubiquitin-like reaction interacts with phosphatidylethanolamine (PE) to produce membrane-bound form of LC3-II, which is attached to the autophagosome membrane, and the expression level of LC3-II is positively correlated with autophagy [25–27]. While Bcl-2 was upregulated, LC3II/I ratio was decreased in the tumors, indicating inhibited apoptosis and autophagy [28]. P62 is one of the autophagy substrate connexins. It has multiple domains. It plays a role by carrying the protein to be degraded into the autophagosome, and it is continuously degraded by the lysosome during the autophagy process [29, 30]. When autophagy is inhibited, p62 can accumulate in cells, and its expression level is negatively correlated with autophagy. In the gene chip assay, P62 gene (DCTN4) expression of ZZXJT group cell was downregulated compared with the untreated cells. This indicated that ZZXJT induced SMMC-7721 cell autophagy. Gene chip results also showed that HIF1A and CCND1 mRNA expression decreased. Including BCL2, they were the downstream gene of JAK/STAT signaling pathway and AKT/mTOR [31–34], too.

## 6. Conclusion

With these findings of the exploration, we conclude that *Zhenzhuxiaoji* decoction induced SMMC-7721 cells apoptosis and autophagy by JAK/STAT and PI3K/AKT/mTOR signaling pathways. Because the target of Chinese medicine is multitudinous, the study will go on.

## Figures and Tables

**Table 1:** Components of ZZXJD.

Chinese name	Name for publishing	Amount (g)	Lot no.	License no.	Place of origin	Company
Nu Zhen Zi	*Ligustrum lucidum Ait., fruit*	15	2104283	Wan 20160095	Anhui, China	Anhui Puren Traditional Chinese Medicine Yinpian Co., Ltd

E Zhu	*Curcuma zedoaria, root*	20	2105121	Wan 20160095	Anhui, China	Anhui Puren Traditional Chinese Medicine Yinpian Co., Ltd

Bai Hua She She Cao	*Hedyotis diffusa*	50	210501	Hei 20160102	Harbin, China	Heilongjiang Deshun Long Traditional Chinese Medicine Yinpian Co., Ltd

Xia Ku Cao	*Prunella vulgaris* L.*,cluster*	30	2106042	Wan 20160095	Anhui, China	Anhui Puren Traditional Chinese Medicine Yinpian Co., Ltd
Gan Cao	*Glycyrrhiza uralensis Fisch.*, *root*	10	20210702	Qian 20180167	Guizhou, China	Guizhou Junzhitang traditional Chinese Medicine Yinpian Co., Ltd

**Table 2:** Primer sequences of qRT-PCR.

Genes	Sequences
*GAPDH*	F: 5′'-GGAGCGAGATCCCTCCAAAAT-3′'
	R:5′'-GGCTGTTGTCATACTTCTCATGG-3′'

*JAK2*	F: 5′'-GTGTGGAGATGTGCCGGTATGAC-3′'
	R: 5′'-GAT TACGCCGACCAGCACTGTAG-3′'

*STAT3*	F: 5′'-CGGAGAAGCATCGTGAG TGAGC-3′'
	R: 5′'GTTGCCTCTTCCAGTCA G-3′'

*AKT*	F: 5′'-GTCATCGAACGCACCTTCCAT-3′'
	R: 5′'-AGCTTCAGGTACTCAAACTCGT-3′'

*mTOR*	F: 5′'-ACCGGCACACATTTGAAGAAG-3′'
	R: 5′'-CTCGTTGAGGATCAGCAAGG-3′'

*LC3*	F: 5′'-AACATGAGCGAGTTGGTCAAG-3′'
	R: 5′'-GCTCGTAGATGTCCGCGAT-3′'

*P62*	F: 5′'-GACTACGACTTGTGTAGCGTC-3′'
	R: 5′'-AGTGTCCGTGTTTCACCTTCC-3′'

*BECLIN1*	F: 5′'-GGTGTCTCTCGCAGATTCATC-3′'
	R: 5′'-TCAGTCTTCGGCTGAGGTTCT-3′'

*BAX*	F: 5′'-CCCGAGAGGTCTTTTTCCGAG-3′'
	R: 5′'-CCAGCCCATGATGGTTCTGAT-3′'

*BCL-2*	F: 5′'-GAACTGGGGGAGGATTGTGG-3′'
	R: 5′'-GCATGCTGGGGCCATATAGT-3′'

**Table 3:** Cell viability of SMMC-7721 cells treated with ZZXJD.

Concentration of drug (%)	n	24 h cell viability (%)	48 h cell viability (%)
0	6	100	100
2.5	6	95.4 ± 3.68	85.1 ± 4.76^Δ^
5.0	6	86.3 ± 4.69^*∗*^	83.7 ± 5.42^Δ^
7.5	6	87.63 ± 6.66^*∗*^	72.5 ± 7.37^Δ^
10.0	6	80.43 ± 6.47^*∗*^	65.9 ± 6.82^Δ^
12.5	6	74.33 ± 9.22^*∗*^	62.9 ± 6.48^Δ^
15.0	6	64.43 ± 6.57^*∗*^	52.4 ± 4.47^Δ^
17.5	6	56.63 ± 1.03^*∗*^	45.8 ± 2.76^Δ^
20.0	6	61.03 ± 3.33^*∗*^	50.6 ± 7.17^Δ^

^*∗*^Compared with the model group, it is statistically significant (*p* < 0.05) in 24 h. ^Δ^Compared with the model group, it is statistically significant (*p* < 0.05) in 48 h. Optimum IC_50_ value of ZZXJD was found to be 17.7% for 48 h incubation. Hence, 17.5% was opted as optimal dose (high-dose) for additional experiment.

**Table 4:** Tumor weight and tumor inhibition rate of nude mice in each group$\left(\bar{x}\pm s\right)$.

Grouping	n	Tumor weight (g)	Tumor inhibition rate (%)
Model group	10	1.161 ± 0.27197	-
Low-dose group	10	0.808 ± 0.20735^ ^*∗*^Δ^	30.40%
Middle-dose group	10	0.726 ± 0.18656^ ^*∗*^Δ^	37.47%
High-dose group	10	0.673 ± 0.19010^ ^*∗*^Δ^	42.05%
Cisplatin group	10	0.565 ± 0.22471^ ^*∗*^^	51.33%

^*∗*^Compared with the model group, it is statistically significant (*p* < 0.05), and ^Δ^is statistically significant compared with cisplatin group (*p* < 0.05).

## Data Availability

The datasets used and/or analyzed during the current study are available from the corresponding author on reasonable request.

## List of Abbreviations

ZZXJD: Zhenzhu Xiaoji decoction
TEM: Transmission electron microscope
AKT: Protein kinase B
mTOR: Mammalian target of rapamycin
JAK2: Janus kinase 2
STAT3: Signal transducer and activator of transcription 3
TCM: Traditional Chinese medicine.
